# Dual protective effect of the association of plant extracts and fluoride against dentine erosion: In the presence and absence of salivary pellicle

**DOI:** 10.1371/journal.pone.0285931

**Published:** 2023-05-18

**Authors:** Samira Helena Niemeyer, Nikola Jovanovic, Sindy Sezer, Lucas Sébastien Wittwer, Tommy Baumann, Thiago Saads Carvalho

**Affiliations:** Department of Restorative, Preventive and Pediatric Dentistry, University of Bern, Bern, Switzerland; International Medical University, MALAYSIA

## Abstract

**Objectives:**

To verify the protective effect of plant extracts associated with fluoride against dental erosion of dentine, in the presence and absence of a salivary pellicle.

**Methods:**

Dentine specimens (n = 270) were randomly distributed into 9 experimental groups (n = 30/group): GT (green tea extract); BE (blueberry extract); GSE (grape seed extract); NaF (sodium fluoride); GT+NaF (green tea extract and NaF); BE+NaF (blueberry extract and NaF); GSE+NaF (grape seed extract and NaF); negative control (deionized water); and a positive control (commercialized mouthrinse containing stannous and fluoride). Each group was further divided into two subgroups (n = 15), according to the presence (P) or absence (NP) of salivary pellicle. The specimens were submitted to 10 cycles: 30 min incubation in human saliva (P) or only in humid chamber (NP), 2 min immersion in experimental solutions, 60 min of incubation in saliva (P) or not (NP), and 1 min erosive challenge. Dentine surface loss (dSL-10 and dSL-total), amount of degraded collagen (dColl) and total calcium release (CaR) were evaluated. Data were analyzed with Kruskal-Wallis, Dunn’s and Mann-Whitney U tests (p>0.05).

**Results:**

Overall, the negative control presented the highest values of dSL, dColl and CaR, and the plant extracts showed different degrees of dentine protection. For the subgroup NP, GSE showed the best protection of the extracts, and the presence of fluoride generally further improved the protection for all extracts. For the subgroup P, only BE provided protection, while the presence of fluoride had no impact on dSL and dColl, but lowered CaR. The protection of the positive control was more evident on CaR than on dColl.

**Conclusion:**

We can conclude that the plant extracts showed a protective effect against dentine erosion, regardless of the presence of salivary pellicle, and that the fluoride seems to improve their protection.

## Introduction

Dental erosion is the loss of dental hard tissues due to the frequent and prolonged contact of acids on the tooth surfaces, not caused by bacteria. It starts with enamel mineral loss and, consequently, a softening of the surface, advancing to a bulk loss of tissue [1]. Its progression results in the exposition of the underlying dentine, which can result in pain (dentin hypersensitivity) and, in turn, a negative impact in the quality of life [1, 2]. The estimated mean global prevalence of dental erosion ranges between 30–50% in deciduous teeth and between 20–45% in permanent teeth [3], with dentine exposure already observed in children and adolescents [4].

Saliva is a natural protection for the teeth. It forms a salivary pellicle composed of proteins, glycoproteins, peptides, lipids and other macromolecules, and it hinders the direct contact of the acids with the tooth surfaces [5], and brings some protection, albeit limited, to the tooth surface [6]. The vast majority of studies on salivary pellicle has focused on their effect on enamel [7], but attention has also been given to the effect of the pellicle on dentine [8–10], where a limited protection has been observed [8]. After exposure to acid, part of the salivary pellicle was still observed on the dentine surface, but the underlying dentine was partially demineralized [9]. Proteomic analyses identified 52 proteins in the dentine salivary pellicle after immersion in deionized water, and after erosion the number was reduced to only 6 proteins [10], indicating that few proteins are acid-resistant.

Currently, many compounds have been tested in an attempt to modify the salivary pellicle and increase its resistance against erosion [11, 12]. Among the compounds tested are polyphenol-rich plant extracts, which have affinity for some salivary proteins [13–15] causing the precipitation and aggregation of these proteins and increasing the thickness of the salivary pellicle. This, in turn, reduces the permeability of the pellicle and increases its acid resistance [16, 17]. Until now, these positive results are mainly for pellicles formed on enamel, but our knowledge regarding pellicles formed on dentine is still very limited.

Investigations on dentine are essential because the organic matrix will greatly influence the outcome [18, 19]. We need to further our insights on how the organic matrix will influence salivary pellicle formation, and this, in turn, will influence dental erosion. More specifically, both the dentine and the saliva contain matrix metalloproteinases (MMPs). These MMPs can get activated under erosive conditions and, consequently, they can degrade the organic matrix, advancing the progression of dentine demineralization [20]. Inhibition of the MMPs will decrease the degradation of the organic matrix and reduce dentine demineralization [21], and this effect can be obtained with some polyphenol-rich plant extracts [22]. Green tea [23, 24], grape seed and cranberry extracts have all shown a protective effect against dentine erosion, probably due to their polyphenol content and to MMP inhibition [25]. Besides, the extracts are also believed to act as cross-linkers; in other words, they can act cross-linking the exposed collagen, thus increasing its resistance to degrading enzymes [21, 26]. In this context, pellicle modification with polyphenol-rich plant extracts should be further investigated, especially in view of dentine biomodification and MMP inhibition.

Likewise, fluoride can also modify the salivary pellicle [27–29] and inhibit MMPs [30–32], possibly leading to a protective, albeit limited, effect. This is in addition to its effect on the mineral, thus, it would be interesting to investigate if there is a synergistic effect between polyphenol-rich plant extracts and fluoride on dentine protection. Therefore, the aim of the present study was to verify the protective effect of plant extracts associated with fluoride against dental erosion on dentine. This was done in the absence and in the presence of a salivary pellicle, to verify, respectively, the effect of the plant extracts and fluoride directly on the dentine surface and on the modification of the dentine salivary pellicle.

## Materials and methods

### Specimen preparation

Human molars were selected from a biobank comprised of teeth extracted by dental practitioners in Switzerland. Before the extraction, the patients were informed about the use of their teeth for research purposes and their oral consent was retrieved. Because of using pooled teeth, the local ethics committee categorized the samples as “irreversibly anonymised”, and no previous ethical approval was necessary. The present experiment was carried out in conformity with the approved guidelines and regulations of the local ethical committee (Kantonale Ethikkommission: KEK).

A total of 270 dentine squares (4x4mm) from the roots of human molars were embedded in acrylic resin (Paladur, Heraeus Kulzer GmbH). All specimens were ground flat with abrasive silicon carbide paper discs of decreasing grain size (18.3 μm to 5 μm) and polished with 3 μm grain size diamond paste (DPStick P, Struers, for 60 s) under constant tap water cooling (TegraPol-15, Struers). The specimens were rinsed with deionized water in an ultrasound bath (60 s) between each step. They were prepared shortly before the beginning of the experiment and kept in tap water at 4°C. Right before start of the experiment, final polishing was performed with 1 μm grain size diamond pastes (DP-Stick P, Struers, for 60s) under constant cooling (TegraPol-15, Struers) and rinsed with deionized water in an ultrasound bath for 60 s. An initial curvature measurement with an optical profilometer (MicroProf 100, FRT the art of metrology) was performed. Then, two thirds of the surface of each dentine specimen was protected with a tape to obtain two reference areas, while the middle third (width about 1.5mm) was let exposed to the treatments.

### Stimulated human saliva collection

Clarified stimulated human saliva was used in the present study. The saliva was collected from adult healthy donors, aged 20–60 years, from both genders, who were informed not to eat or drink (except water) for at least 60 min before collection. Donors who smoked or used fixed orthodontic devices were excluded from the collection. The local ethical committee (KEK) considers pooled samples saliva as irreversibly anonymized samples, so prior ethical approval was not required. The saliva collection was performed in the morning, always at the same time. Fifteen donors chewed on paraffin (Paraffin pellets, Ivoclar Vivadent) for 10 min and collect saliva into ice-chilled vials. The stimulated saliva from all donors was immediately pooled, centrifuged (3’000 g, 15 min, 4°C) and the supernatant fraction was collected.

To avoid degradation of the salivary proteins, protease inhibitors (phenylmethanesulfonyl fluoride; n-ethylmaleimide; 1,10-phenanthroline monohydrate) were added to the previously prepared human saliva. These inhibitors were initially dissolved in methanol (400 mM) and then compounded till a final concentration of 100 mM per inhibitor. The solution containing all inhibitors was added to the clarified human saliva (1:100). Then, aliquots were prepared and stored at -80°C until the beginning of the experiment.

### Experimental procedures and dentine surface loss

The dentine specimens were divided into 9 experimental groups: GT (green tea extract); BE (blueberry extract); GSE (grape seed extract); NaF (500 ppm fluoride, as sodium fluoride—NaF); GT+NaF (green tea extract and 500 ppm fluoride); BE+NaF (blueberry extract and 500 ppm fluoride); GSE+NaF (grape seed extract and 500 ppm fluoride); negative control (deionized water); and a positive control (commercialized mouthrinse: elmex^®^ Erosionsschutz, 800 ppm stannous, as stannous chloride, and 500 ppm fluoride, as NaF and amino fluoride—AmF). Each of these groups contained 30 dentine specimens. Later, they were divided into two subgroups (n = 15/subgroup): one with salivary pellicle (P) and another without salivary pellicle (NP).

For the subgroups P, an aliquot of freshly thawed human saliva containing protease inhibitors was placed on the dentine specimens for 30 min at 37°C, enabling initial protein adherence and initial pellicle formation on the dentine surface. Then, the specimens were immersed in one of the experimental solutions for 2 min at 25°C. Afterwards a new fresh aliquot of saliva was placed on the specimens for a further 60 min at 37°C, for an additional pellicle formation. For the subgroups NP, the specimens remained in a humid chamber at 37°C for 30 min and, subsequently were incubated in one of the experimental solutions, as described for subgroup P. Then, they stayed a further 60 min in the humid chamber (37°C).

Afterwards, the specimens were individually submitted to an erosion challenge, consisting of immersion in 1% citric acid (pH 3.6, 25°C) for 1 min, under constant movement (70 rpm, travel path 50 mm; shaking water bath, SBS40, Stuart). The citric acid was kept for analysis of calcium released by the dentine specimens. This experimental sequence was repeated 10 times. Following the 10th cycle, the tapes were removed, and the same area of the dentine surface was scanned with the optical profilometer (MicroProf 100, FRT the art of metrology), under humidity control.

Dentine surface loss (dSL-10) was determined by subtracting the mean height of the exposed area (1.5 mm) from the mean height of both reference areas, using a specific software (FRT Mark III, FRT the art of metrology, Germany). Initial values (which correspond to the initial surface curvature) were subtracted from the final analyses, to counterbalance the initial surface curvature of each specimen.

Next, the dentine specimens were again protected with the adhesive tape, which were placed on the same reference areas as previously. The demineralized organic matrix of all specimens was removed by immersing them individually in a saline solution containing type VII collagenase from the gram-positive bacterium Clostridium histolyticum (100 U/ml; C0773, Sigma-Aldrich) for 96 h at 37°C and constant movement (70 rpm). Subsequently, the tapes were removed, and the same dentine surface area was re-measured with the optical profilometer (MicroProf 100, FRT the art of metrology) as previously. The total dentine surface loss (dSL-total) was determined. The amount of degraded collagen (dColl) was calculated as the difference between dSL-10 and dSL-total.

### Calcium released to the citric acid

To quantify dentine mineral loss, the calcium released to the citric acid after the erosive cycles was measured. For the total calcium release, 1 ml of citric acid after each erosive cycle was mixed per specimen and analyzed with an atomic absorption spectrometer (Aanalyst 400, Perkin-Elmer Analytical Instruments). The measured amount of calcium was normalized to the exposed dentine surface area [33]. The later was measured using a light microscope connected to a camera (Leica, M420 and Leica DFC495, respectively) under 20x magnification and a software program (IM500). The total amount of calcium release (CaR) per specimen was estimated considering all 10 erosive cycles.

### Statistical analysis

Data were evaluated with Shapiro-Wilk normality test and due to lack of normal distribution of some groups, non-parametric tests were used. Kruskal-Wallis for comparison between groups, considering each subgroup (with and without pellicle) separately, was performed for each response variable: dentine surface loss after 10 cycles (dSL-10), total dentine surface loss (dSL-total), amount of degraded collagen (dColl) and calcium released to the citric acid (CaR). Post hoc Dunn’s test with p corrected for multiple comparisons was performed. Each group was analyzed with Mann-Whitney U test considering the subgroups (P and NP). Moreover, the effect of fluoride was analyzed with Mann-Whitney U test for each plant extract, and for the controls (deionized water and fluoride). Significance level was set at 5% and the analyses were performed with GraphPad Prism 7 for Mac.

## Results

### Dentine surface loss–Subgroup without salivary pellicle (NP)

**Fig 1:**
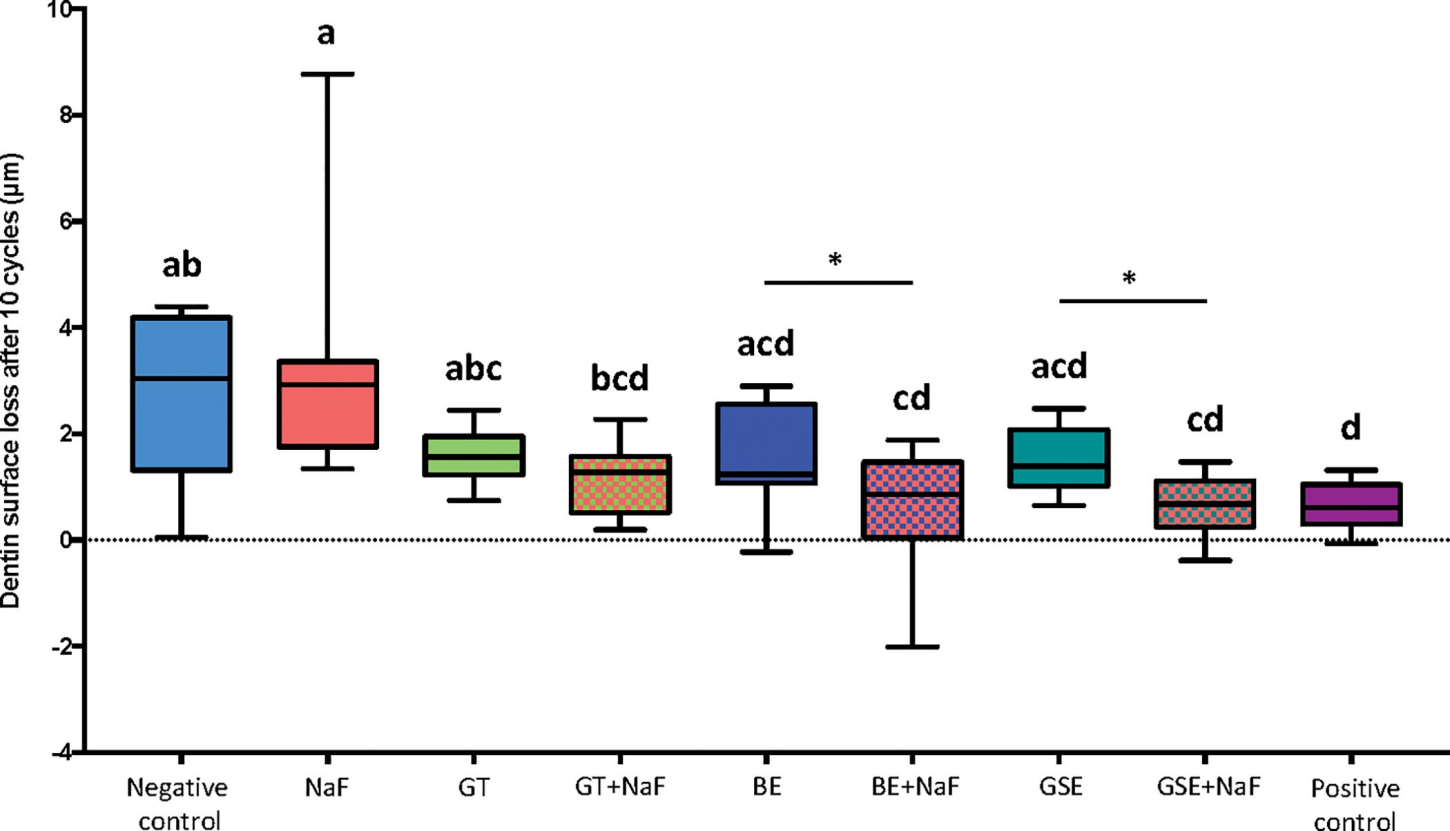
Dentine surface loss after 10 erosive cycles for the groups without salivary pellicle (NP). Different letters denote significant differences between the experimental groups. * Denotes significant effect of adding fluoride to the plant extracts, for the pair comparison for the factor presence of fluoride.

Fig 1 shows the results of dentine surface loss after 10 cycles (dSL-10) for the subgroup NP. The negative control and NaF showed the highest values of dentine surface loss, differing significantly from BE+NaF, GSE+NaF and the positive control. These three groups presented the lowest values of dentine surface loss after 10 erosive cycles. When considering the factor “fluoride”, the presence of fluoride reduced significantly the dentine surface loss for the groups BE and GSE. Negative control and NaF were not significantly different (p = 0.870).

**Fig 2:**
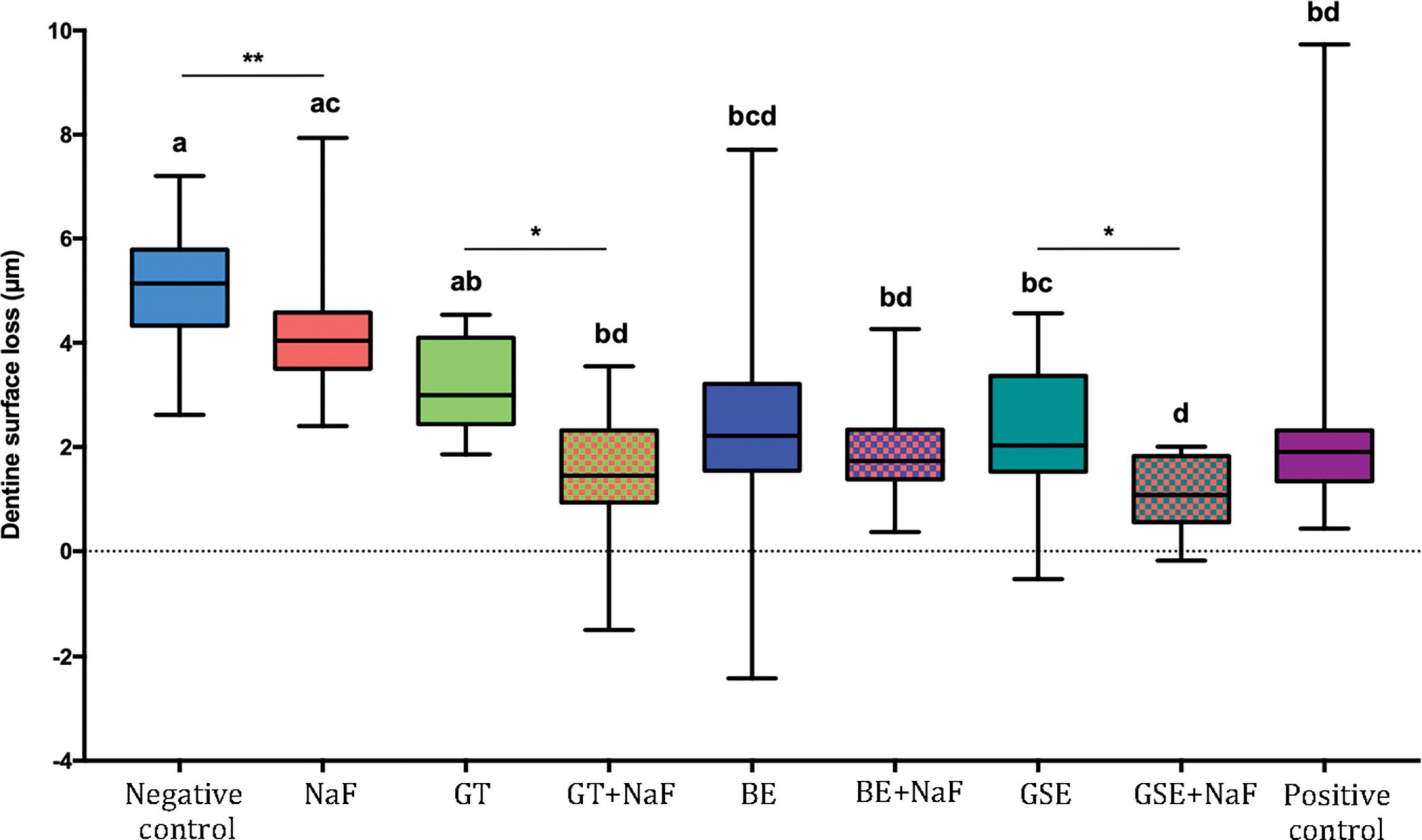
Dentine surface loss after 10 erosive cycles and removal of degraded collagen for the groups without salivary pellicle (NP). Different letters denote significant differences between the experimental groups. ** Denotes significant difference between Negative control and NaF groups, for the pair comparison for the factor presence of fluoride. * Denotes significant effect of adding fluoride to the plant extracts, for the pair comparison for the factor presence of fluoride.

**Fig 3:**
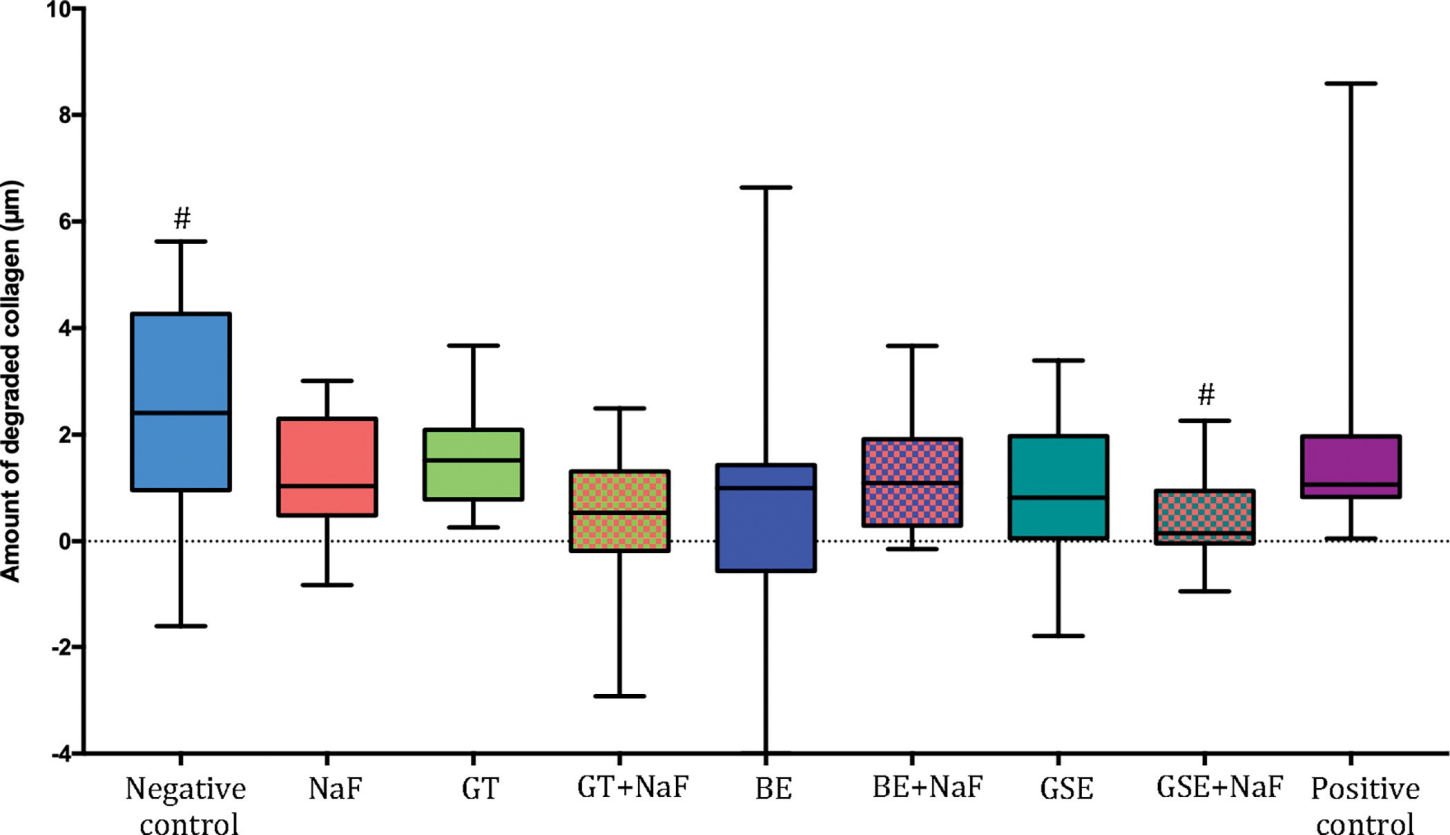
Amount of degraded collagen after 10 erosive cycles for the groups without salivary pellicle (NP). ^#^ Denotes significant difference between Negative control and GSE+NaF.

After removal of the degraded collagen, the dentine surface loss (dSL-total) increased, as shown in Fig 2 for the subgroup NP. The negative control showed the highest values of dentine surface loss, not differing significantly from NaF and GTE. The group GSE+NaF presented the lowest values of dentine surface loss, not differing from the positive control and from GT+NaF, BE and BE+NaF. When considering the factor “fluoride”, the presence of fluoride reduced significantly the dentine surface loss for the groups GTE and GSE. Negative control showed significantly higher surface loss than NaF for the pair comparison (p = 0.021). Regarding the amount of degraded collagen (Fig 3), significant differences between groups were only observed for the negative control and GSE+NaF (p = 0.012).

### Dentine surface loss–Subgroup with salivary pellicle (P)

**Fig 4:**
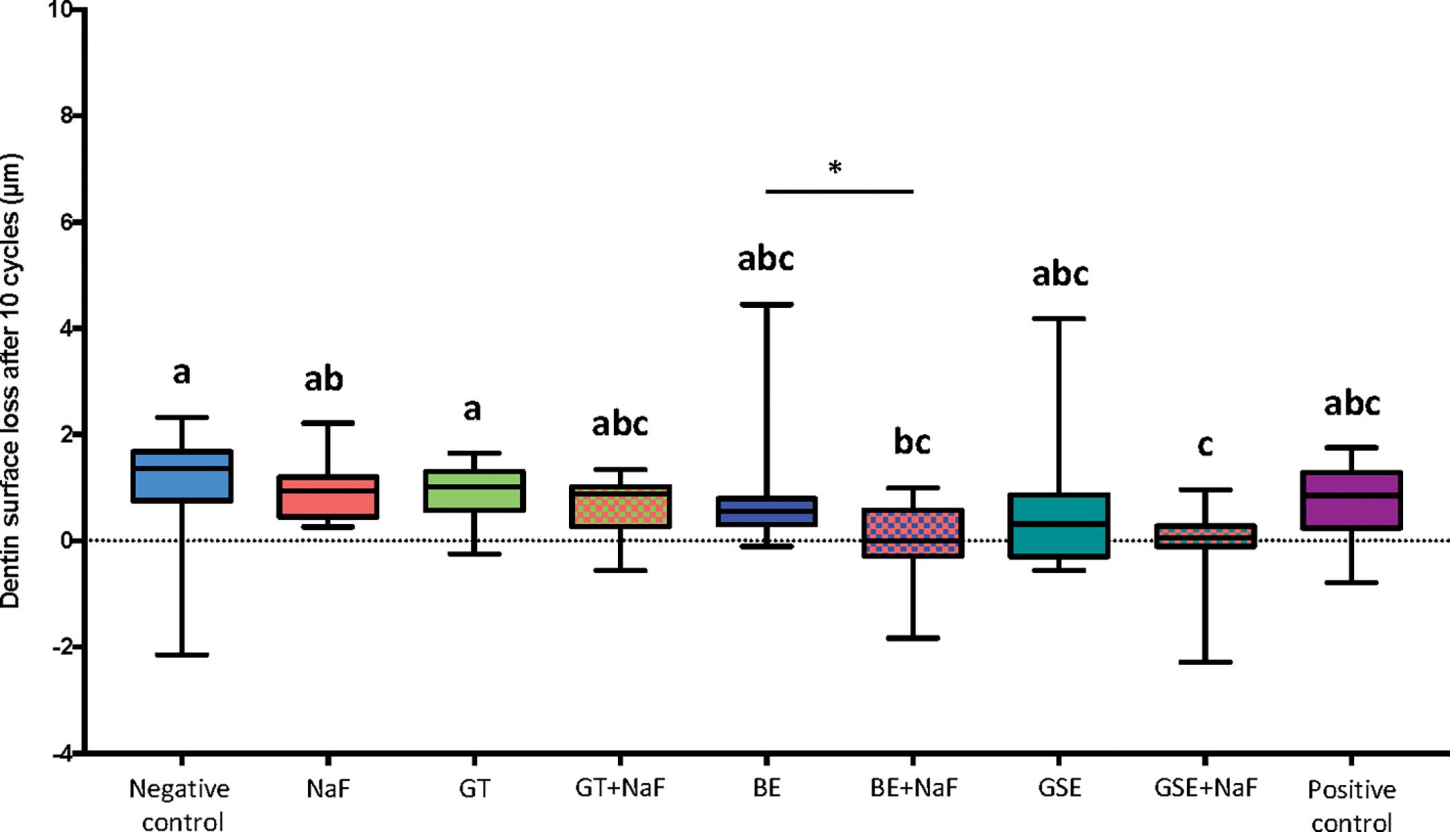
Dentine surface loss after 10 erosive cycles for the groups with salivary pellicle (P). Different letters denote significant differences between the experimental groups. * Denotes significant effect of adding fluoride to the plant extracts, for the pair comparison for the factor presence of fluoride.

Fig 4 shows the results of dentine surface loss after 10 cycles (dSL-10) for the subgroup P. The negative control showed the highest values of dentine surface loss, differing significantly from BE+NaF and GSE+NaF, which were the groups with the lowest values of surface loss. The positive control was not significantly different from the negative control nor the NaF. When considering the factor “fluoride”, the presence of fluoride reduced significantly the dentine surface loss only for the BE group. Negative control and NaF were not significantly different (p = 0.161).

**Fig 5:**
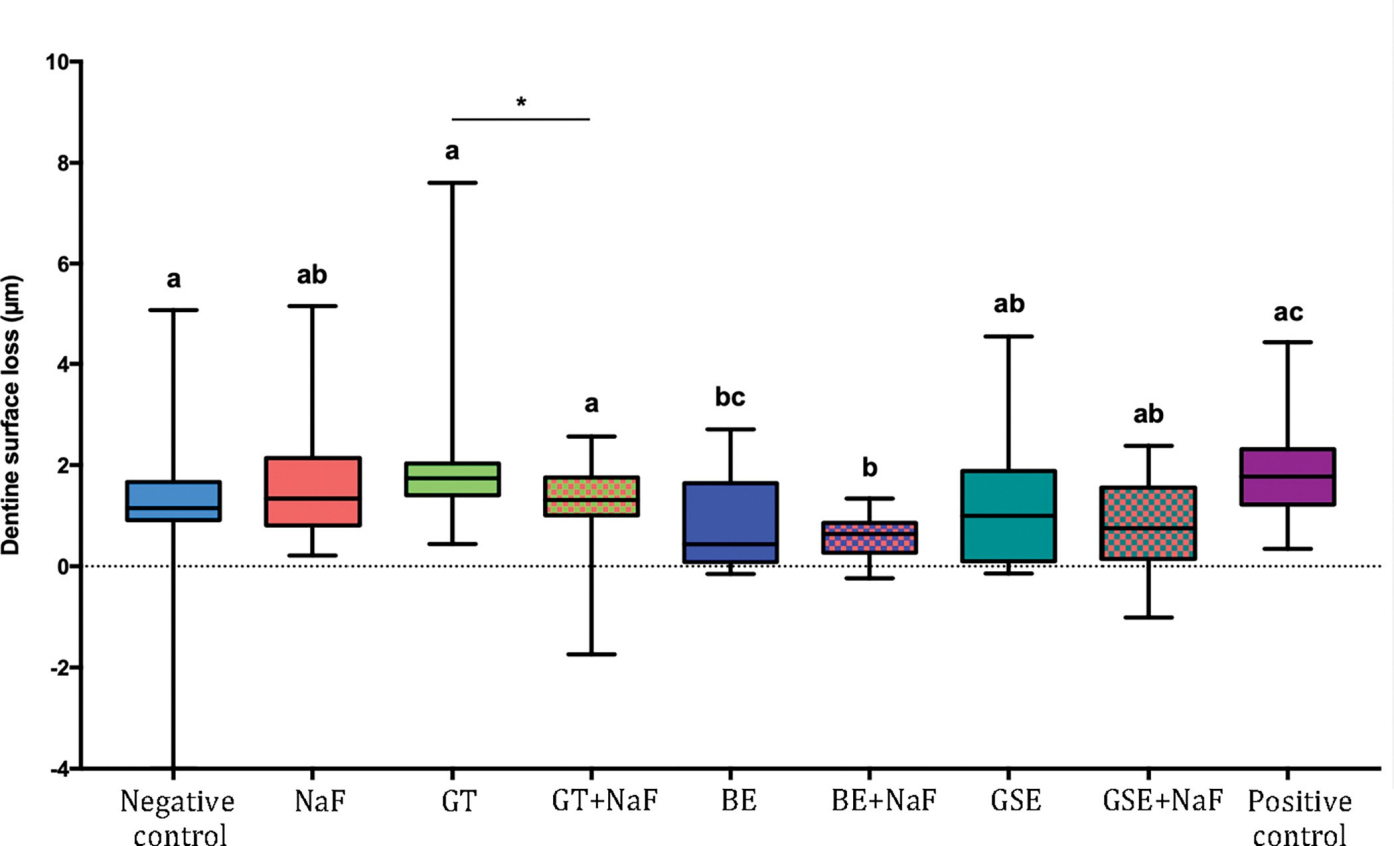
Dentine surface loss after 10 erosive cycles and removal of degraded collagen for the groups with salivary pellicle (P). Different letters denote significant differences between the experimental groups. * Denotes significant effect of adding fluoride to the plant extracts, for the pair comparison for the factor presence of fluoride.

**Fig 6:**
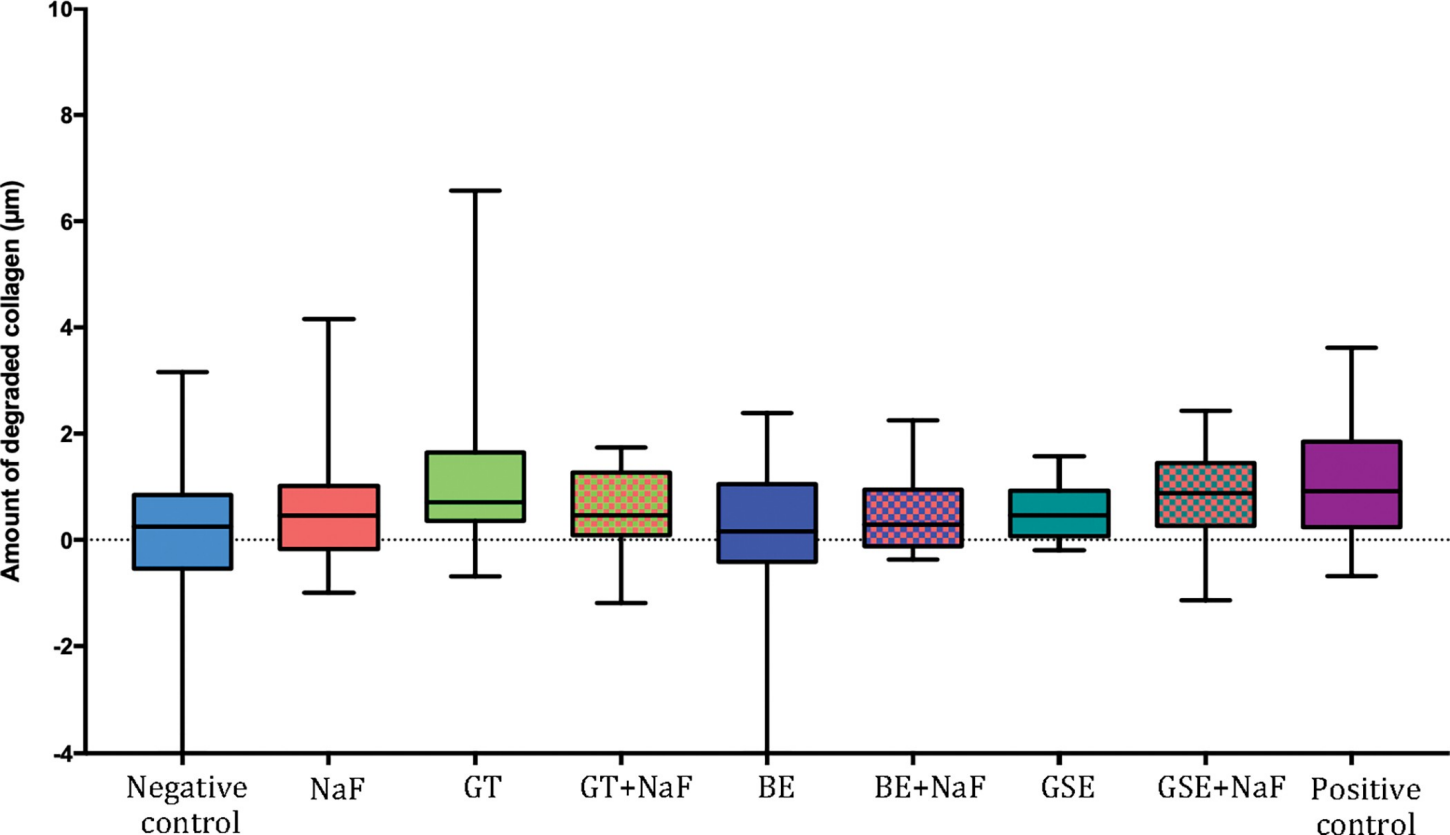
Amount of degraded collagen after 10 erosive cycles for the groups without salivary pellicle (NP). No significant difference was observed between experimental groups.

For the subgroup P, the removal of degraded collagen did not seem to increase the amount of dentine surface loss. Fig 5 shows the results of dentine surface loss after collagen removal (dSL-total) for the subgroup P. BE+NaF was the group with the lowest surface loss. However, it did not differ significantly from NaF, BE, GSE and GSE+NaF. When considering the factor “fluoride”, the presence of fluoride reduced significantly the dentine surface loss only for the group GTE (p = 0.037), although this difference was not significant in all groups comparison (p>0.05). Regarding the amount of degraded collagen (Fig 6), no significant difference was observed.

### Dentine surface loss–Comparison of both subgroups without (NP) and with salivary pellicle (P)

When comparing the effect of the pellicle for each treatment group on dentine surface loss after 10 cycles (Figs 1 and 4), the presence of salivary pellicle significantly decreased surface loss for all groups excepted for the positive control, which did not present significant difference (p = 0.533). Regarding the effect of the pellicle for each treatment group on total dentine surface loss (Figs 2 and 5), significant differences were observed for the negative control, GTE, BE, BE+NaF and GSE, where the presence of pellicle showed lower loss than without pellicle. For amount of degraded collagen (Figs 3 and 6), only the deionized water group showed significant difference between P and NP, the later presenting higher amount of degraded collagen than P.

### Calcium released to the citric acid–Subgroup without salivary pellicle (NP)

Dentine mineral loss was analyzed by measuring the calcium released to the citric acid and is presented as nanomole of calcium per mm2 of exposed dentine surface.

**Fig 7:**
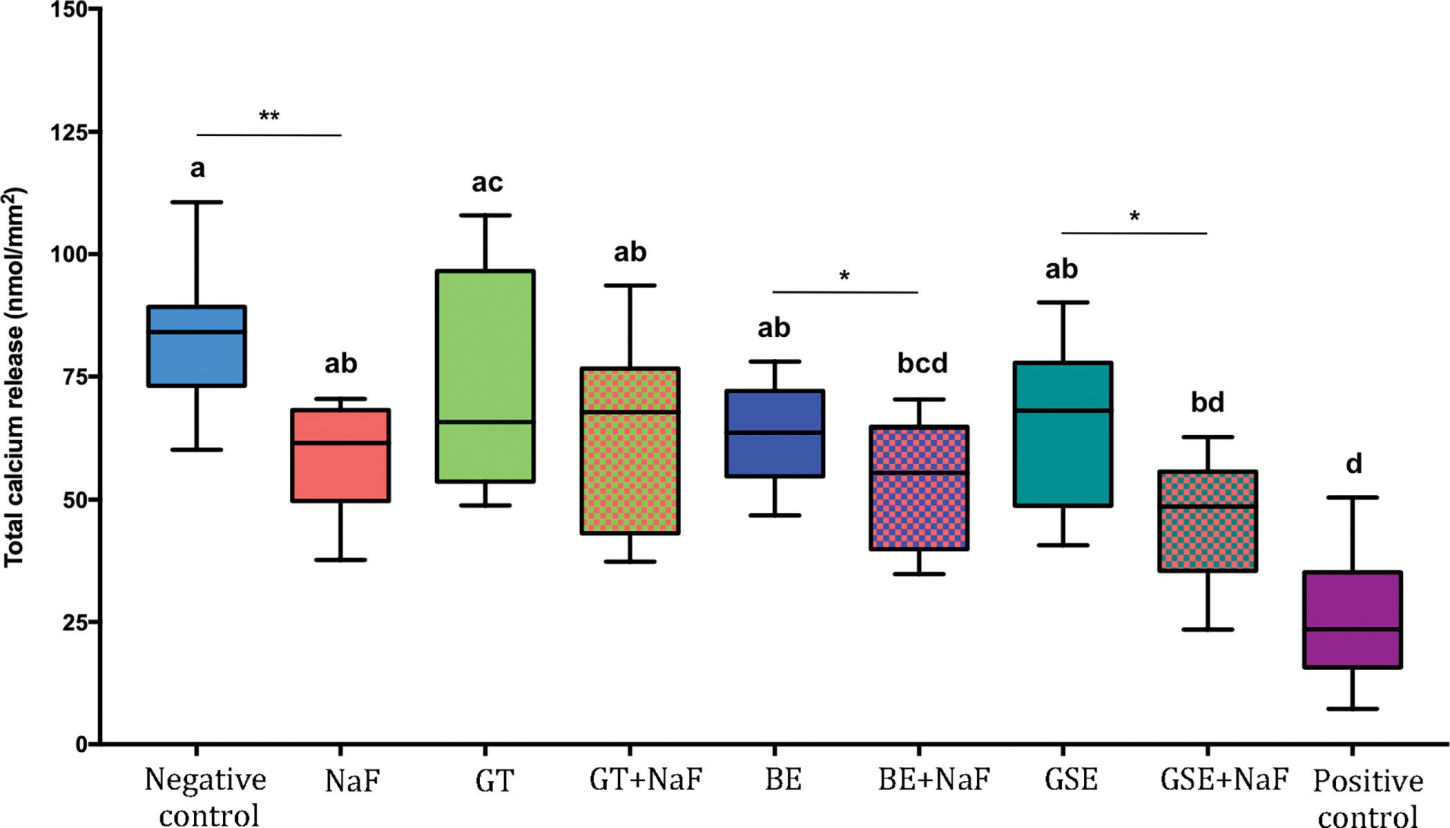
Total calcium release after 10 erosive cycles for the groups without salivary pellicle (NP). Different letters denote significant differences between the experimental groups. ** Denotes significant difference between Negative control and NaF groups, for the pair comparison for the factor presence of fluoride. * Denotes significant effect of adding fluoride to the plant extracts, for the pair comparison for the factor presence of fluoride.

Fig 7 shows the results of total calcium release for the subgroup NP. Negative control showed the highest values of calcium release, not differing significantly from GTE, GTE+NaF, BE and GSE. When considering the factor “fluoride”, the presence of fluoride reduced significantly the amount of calcium release for the groups BE and GSE. Negative control showed significantly higher surface loss than NaF for the pair comparison (p<0.001).

### Calcium released to the citric acid–Subgroup with salivary pellicle (P)

**Fig 8:**
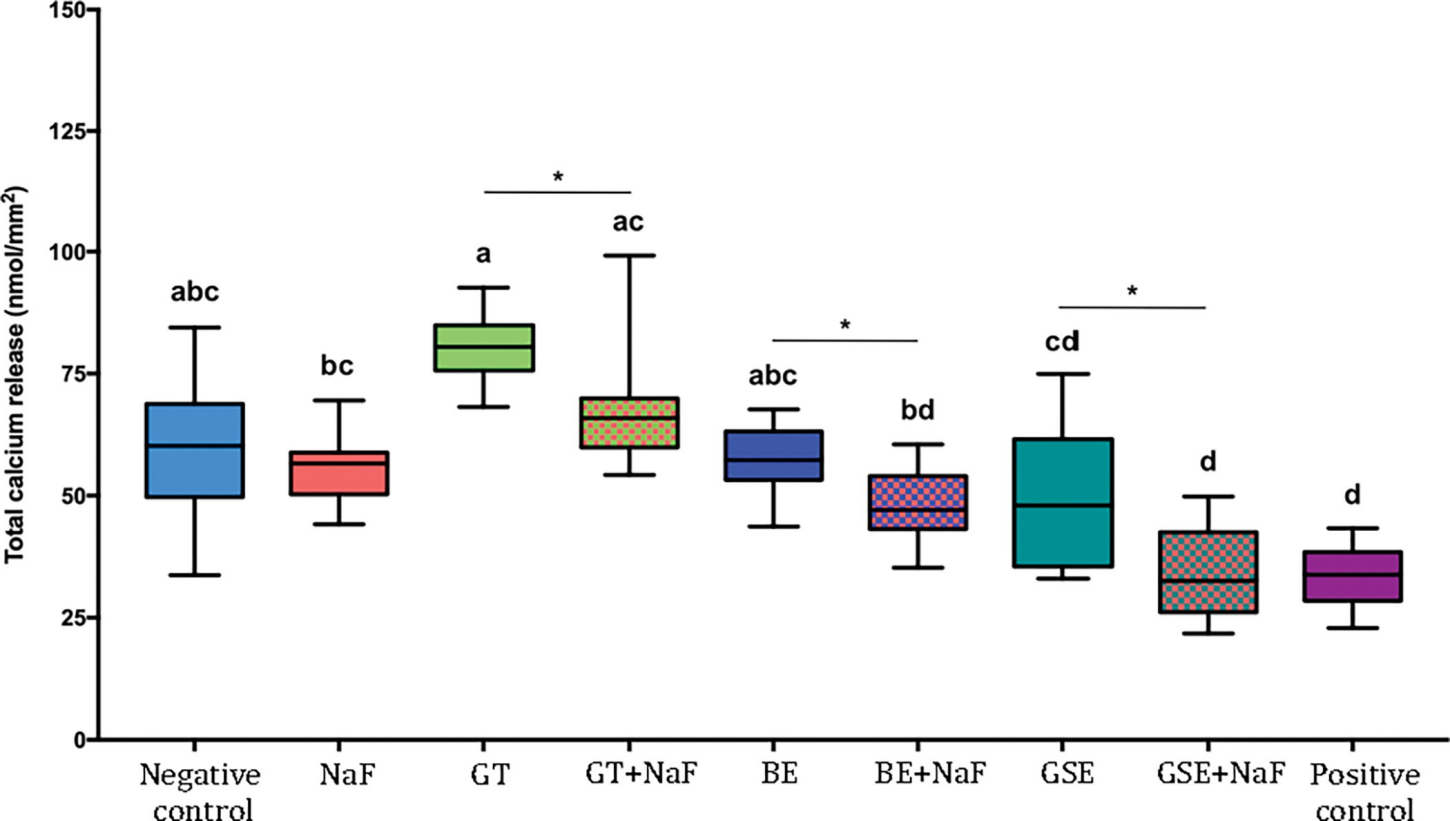
Total calcium release after 10 erosive cycles for the groups with salivary pellicle (P). Different letters denote significant differences between the experimental groups. * Denotes significant effect of adding fluoride to the plant extracts, for the pair comparison for the factor presence of fluoride.

In the presence of salivary pellicle (P, Fig 8), GTE and GTE+NaF presented the highest values of calcium release, without difference between each other and to the negative control. The group NaF was not significantly different than the negative control, neither for the pair comparisons considering the factor “fluoride” (p = 0.461). In this case, the presence of fluoride reduced significantly the calcium release for all plant extracts: GTE (p<0.001), BE (p = 0.001), and GSE (p = 0.002).

### Calcium released to the citric acid–Comparison of both subgroups without (NP) and with salivary pellicle (P)

Irrespective of pellicle formation (P or NP), lowest values of calcium release were observed for positive control, without difference to GSE+NaF. When considering the factor presence of salivary pellicle, comparisons within each group showed significant differences for groups GSE, GSE+NaF, negative control and positive control. For groups GSE, GSE+NaF and negative control, the presence of pellicle (P) significantly reduced calcium release when compared to NP. As for positive control, higher calcium release was observed for P than for NP.

## Discussion

Health benefits from polyphenol-rich plants include protection in the oral cavity, since the polyphenols can interact with salivary proteins from the pellicle, and they can protect against caries [34] and erosion [17, 35]. Several studies have already shown the benefits of polyphenols against erosion, and more specifically on dentine. The present study further explored the effect on these polyphenol-rich plant extracts, namely green tea, blueberry and grape seed, on the dentine and on the dentine pellicle, as well as the effect of fluoride, on the protection against dentine erosion. These extracts were chosen because of their type of polyphenols and because our findings reveal a positive effect of these polyphenols on pellicle modification for enamel protection [36] and against dentine erosion. As already described by Niemeyer et al. [37], this protection mechanism can be three-fold: i) interacting with the dentine (salivary) pellicle, ii) interacting with the collagen of the dentine itself, iii) inhibiting the proteases, such as the matrix metalloproteinases (MMPs), both from the saliva and the dentine.

Regarding the first mechanism of action (interacting with dentine pellicle), one must focus on the molecular structure of the polyphenols. They have a basic chemical structure of phenol rings and galloyl esters or hydroxyl groups. These moieties can form complexes with salivary proteins, especially proline-rich proteins (PRP) [38], and statherin [15]. These proteins are especially present in the basal layer of the pellicle, so it is reasonable to assume that the polyphenol molecules will interact with this basal layer, aggregating salivary proteins and attracting even more proteins to the pellicle, ultimately changing its structure and increasing its thickness [17]. This thicker pellicle can therefore have an increased protective effect against erosion [39, 40]. Furthermore, since polyphenols attract more proteins to the pellicle, they are capable of changing the proteomic profile of the salivary pellicle [41]. Our results also suggest this occurred with the polyphenols chosen for this study.

Previous studies have already shown the positive effects of green tea against erosion [23, 36, 42–44]. We similarly observed positive results. This is probably because green tea extract is obtained from Camelia sinensis leaves, which is rich in polyphenols called catechins, especially epigallocatechin-3-gallate (EGCG). Other studies have already shown that EGCG can cross-link proteins in the pellicle, leading to a thicker, more electron-dense pellicle [39, 40], containing more acid-resistant proteins, such as statherin [41].

Regarding the grape seed extract, it mainly contains purified oligomeric proanthocyanidin (OPC). This polyphenol has catechin and epicatechin units with partial galloylation [45], and they can form hydrophobic interactions or hydrogen bonds with proteins, or even oxidation followed by covalent condensations [14, 38, 46]. So, these molecules will easily bind to pellicle proteins, especially to PRP and statherin [15]. We therefore speculate that OPC acts in a similar way to EGCG, where it interacts with the basal layer of the pellicle, attracting even more proteins to the pellicle, increasing its thickness and, ultimately, changing its structure and improving its protective effect [39, 40]. The protective effect of Grape seed extract on the pellicle had already been observed for pellicles formed on enamel [36], but not yet for pellicles formed on dentine. Now our results support the effect of grape seed extract also on pellicles formed on dentine.

Regarding the second and third mechanisms of action (interacting with the collagen layer and inhibiting the MMPs), one must understand the erosion process on dentine. This process is particularly affected by the organic matrix, in other words, the collagen layer in dentine. With acid attacks, the dentine surface demineralizes, causing an initial loss of its mineral component. With increasing attacks, the demineralized layer exposes the organic component of the dentine. The amount of exposed collagen layer increases as the erosion process progresses, but when this layer reaches a certain thickness, it acts as barrier against further acid attacks, and the demineralization rate decreases. However, this layer can be degraded by MMPs present in saliva or in the dentine itself. If the collagen layer is degraded by the MMPs, the mineral part of the dentine will be further exposed to the acids and the demineralization will progress. So, if the collagen layer is cross-linked and preserved, and if the MMPs are also inhibited from degrading the collagen, this organic layer will remain on the surface of the dentine for longer, protecting it against further demineralization.

The EGCG from green tea has the galloyl moiety, and this can change the conformation of the MMPs, thus inhibiting the proteases and hindering their effect on the collagen [47]. EGCG is further able to chelate the zinc atoms present in the MMPs, which is another mechanism of inhibiting these proteases from degrading the collagen [48–51]. Although EGCG inhibits MMPs, our results rather suggest that these molecules will act on the collagen layer. The galloyl moiety in EGCG contains many hydroxyl groups, which can form more hydrogen bonds with the collagen in the organic layer of the dentine, and this, in turn, improves the mechanical properties of the collagen, reducing its degradation [47], and reducing erosive demineralization of the dentine.

Likewise, the grape seed extract can also protect the dentine against surface loss by its effect on the collagen and the MMPs. The OPC can bind to the organic layer in the dentine, they are also known natural cross-linking agents that easily bind to the collagen fibers [52] and hinder their degradation, improving the mechanical properties of the dentine surface. This is in agreement with other studies [25, 53].

When observing the two subgroups of our study (solutions with and without fluoride), we observed generally a significant better protection in the solutions where fluoride was present. The protection of fluoride against erosion is rather believed to be related to the formation of CaF2-like particles on the tooth surface, which serve as a physical barrier against the acid challenges [54]. This depends on the fluoride concentration and pH of the product. However, even under optimized conditions, the formation of CaF2-like particles does not completely cover the tooth surface, and the protection from monovalent fluoride products in erosion prevention might be of limited outcome [55, 56]. The simultaneous effect, however, of fluoride with the polyphenols lead to increased protection against erosion. Our results, therefore suggest that the solutions containing both active ingredients will act both on pellicle modification [35], because they cross-link the proteins and increase the protein-protein interaction, eventually leading to a more electron-dense and thicker pellicle, on the collagen layer of the dentine, by crosslinking the fibers and inhibiting the MMPs, and the fluoride can act on the mineral layer of the dentine, forming more CaF2-like particles.

We therefore conclude that the plant extracts showed a protective effect against dentine erosion, regardless of the presence of salivary pellicle, and that the fluoride seems to improve their protection. Future studies should test this combination of plant extracts and fluoride using in situ models. Besides, these compounds could be formulated using a composition more closely to a commercial mouthrinse or even as active ingredients in toothpastes. Furthermore, the impact of brushing challenges would also be of interest. Finally, proteomics studies could be conducted to gain further insight into the exact mechanism of the protection provided through the treatment with these compounds.

## Supporting information

S1 Data (XLSX)
